# Hyperspectral imaging for small-scale analysis of symptoms caused by different sugar beet diseases

**DOI:** 10.1186/1746-4811-8-3

**Published:** 2012-01-24

**Authors:** Anne-Katrin Mahlein, Ulrike Steiner, Christian Hillnhütter, Heinz-Wilhelm Dehne, Erich-Christian Oerke

**Affiliations:** 1 Institute for Crop Science and Resource Conservation (INRES) - Phytomedicine, University of Bonn, Nussallee 9, 53115 Bonn, Germany

**Keywords:** hyperspectral imaging, spectral reflectance, plant disease, leaf traits, *Cercospora beticola*, *Erysiphe betae*, *Uromyces betae*

## Abstract

Hyperspectral imaging (HSI) offers high potential as a non-invasive diagnostic tool for disease detection. In this paper leaf characteristics and spectral reflectance of sugar beet leaves diseased with *Cercospora *leaf spot, powdery mildew and leaf rust at different development stages were connected. Light microscopy was used to describe the morphological changes in the host tissue due to pathogen colonisation. Under controlled conditions a hyperspectral imaging line scanning spectrometer (ImSpector V10E) with a spectral resolution of 2.8 nm from 400 to 1000 nm and a spatial resolution of 0.19 mm was used for continuous screening and monitoring of disease symptoms during pathogenesis. A pixel-wise mapping of spectral reflectance in the visible and near-infrared range enabled the detection and detailed description of diseased tissue on the leaf level. Leaf structure was linked to leaf spectral reflectance patterns. Depending on the interaction with the host tissue, the pathogens caused disease-specific spectral signatures. The influence of the pathogens on leaf reflectance was a function of the developmental stage of the disease and of the subarea of the symptoms. Spectral reflectance in combination with Spectral Angle Mapper classification allowed for the differentiation of mature symptoms into zones displaying all ontogenetic stages from young to mature symptoms. Due to a pixel-wise extraction of pure spectral signatures a better understanding of changes in leaf reflectance caused by plant diseases was achieved using HSI. This technology considerably improves the sensitivity and specificity of hyperspectrometry in proximal sensing of plant diseases.

## Background

The reflectance of leaves is the result of multiple interactions between incoming irradiation and biophysical (e.g. leaf surface, tissue structure) and biochemical characteristics (e.g. content of pigments and water) of plants [1-3]. Several studies have described the prospects of sensing leaf reflectance in the visible (VIS, 400-700 nm), near infrared (NIR, 700-1000 nm) and short wave infrared (SWIR, 1000-2500 nm) for detecting changes in plant vitality with emphasis on fungal plant diseases using non-imaging spectroradiometers [4-7]. Disease symptoms result from physiological changes in plant metabolism due to activities of pathogens [8]. The impact on physiology and phenology of plants varies with the type of host-pathogen interaction and may cause modifications in pigments, water content, and tissue functionality of plants or in the appearance of pathogen-specific fungal structures [9,10]. All these factors may change the spectral characteristics of plants. Knowledge on the effects of pathogens on the metabolism and structure of plant tissue is therefore essential for hyperspectral discrimination of healthy and diseased leaf and canopy elements [11].

Hyperspectral imaging is an innovative technology with high potential for non-invasive sensing of the physiological status of vegetation [12-14] and may allow an objective and automatic assessment of the severity of plant diseases in combination with continuative data analysis methods [15]. Further to spectral information from non-imaging spectroradiometers, hyperspectral cameras enable the detection of spectral and spatial information of objects of interest. Hyperspectral imaging is expected to improve disease detection through a better examination of host-pathogen interactions [15,16]. Imaging sensor systems allow a pixel-wise attribution of disease-specific symptoms and healthy tissue and improve both, the specificity and sensitivity of disease detection by technical sensors [13].

In most studies using hyperspectral imaging, the spectral signature of tissue colonized by a pathogen is compared to the spectral signature of healthy tissue and plant canopies. Bravo *et al. *[17] used in-field spectral images for an early detection of yellow rust in wheat, Nansen *et al. *[18] analyzed hyperspectral data cubes for the detection of insect-induced stress in wheat plants, and Polder *et al. *[19] combined different optical sensors for the detection of tulip breaking virus. Hyperspectral imaging has recently become more common in monitoring of the quality and security of fruits and food. Balasundaram *et al. *[20] and Qin *et al. *[21] developed a hyperspectral imaging approach to detect canker lesions on citrus fruits.

Disease assessment of plants by technical sensors may be differentiated into detection (i.e. deviation from healthy), identification (i.e. diagnosis of specific symptoms among others, differentiation of various diseases) and quantification (i.e. measurement of disease severity, e.g. percentage leaf area affected). The type and amount of sensor information vary with the objective. Sensors have to be sensitive to the effect of fungal colonization of plant tissue during pathogenesis [8].

The advancement from non-imaging spectroradiometry to hyperspectral imaging enables the pixel-wise attribution of spectral signatures suitable for the assessment of modifications on a small scale, typical for early stages of plant diseases. Significant changes in spectral signature of host tissue may be detected not only for the tiny spots of primary symptoms and during further disease stages, but also for localized effects in pre-symptomatic stages and different effects on plant tissue. This information may be linked to fundamental processes of plant biology and fungal pathogenesis.

The potential of hyperspectral imaging for small-scale analysis of symptoms of plant diseases was explored using three foliar diseases of sugar beet as a model system. *Cercospora *leaf spot (CLS), powdery mildew (PM) and sugar beet rust (SBR) are caused by the fungal pathogens *Cercospora beticola *(Sacc.), *Erysiphe betae *(Vanha) Weltzien and *Uromyces betae *(Persoon) Lev., respectively. Spectral signatures of disease-specific symptoms in different developmental stages and of different regions of typical symptom were evaluated on pixel basis. The detection, differentiation and quantification of diseases were realized using an automatic classification algorithm. Morphological changes of the leaves due to pathogen colonisation were described microscopically and leaf structure was linked to spectral reflectance patterns.

## Results

### Effect of fungal colonization on structure and reflectance of leaves

**Figure 1:**
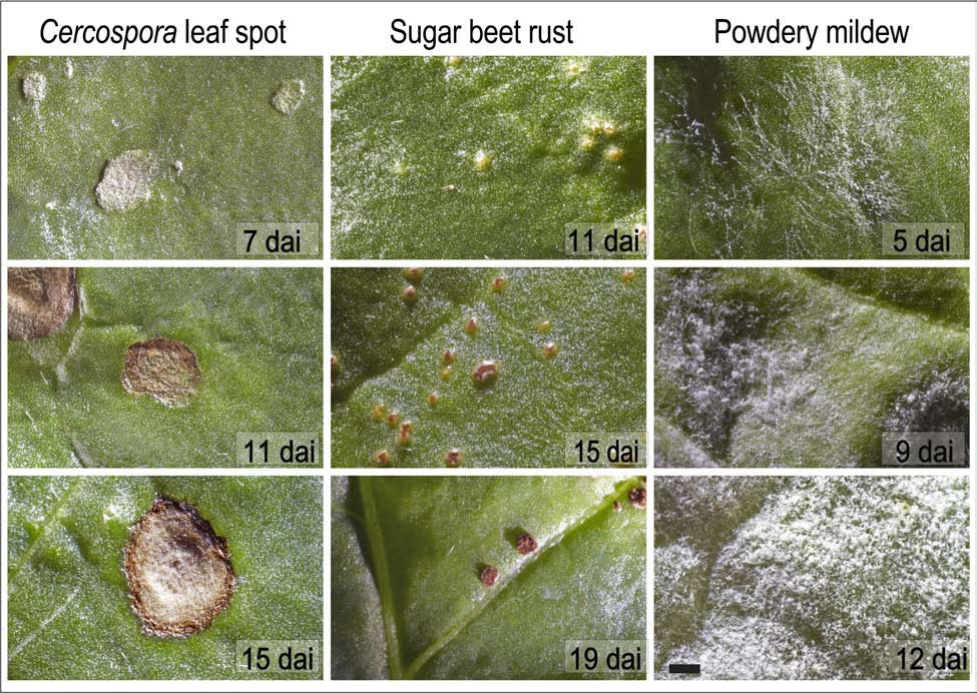
**Disease specific symptoms of *Cercospora *leaf spot 7, 11, and 15 dai (A, D, G), sugar beet rust 11, 15, and 19 dai (B, E, H) and powdery mildew 5, 9, and 12 dai (C, F, I) at different stages of pathogenesis (scale bar denotes 2000 μm)**.

Symptoms differed between the foliar diseases and within their developmental stages during pathogenesis. First symptoms of CLS were small grey and sunken spots (Figure 1A). With on-going pathogenesis these spots became necrotic and a reddish-brown margin was formed (Figure 1D, G). Primary symptoms of SBR were tiny chlorotic spots (Figure 1B). At later stages the epidermis is ruptured by amber uredospores (Figure 1E, H). The first symptoms of PM are small mycelial colonies on the upper side of sugar beet leaves (Figure 1D). These colonies expand rapidly over the leaf surface and mycelial density increased during pathogenesis (Figure 1F, I).

**Figure 2:**
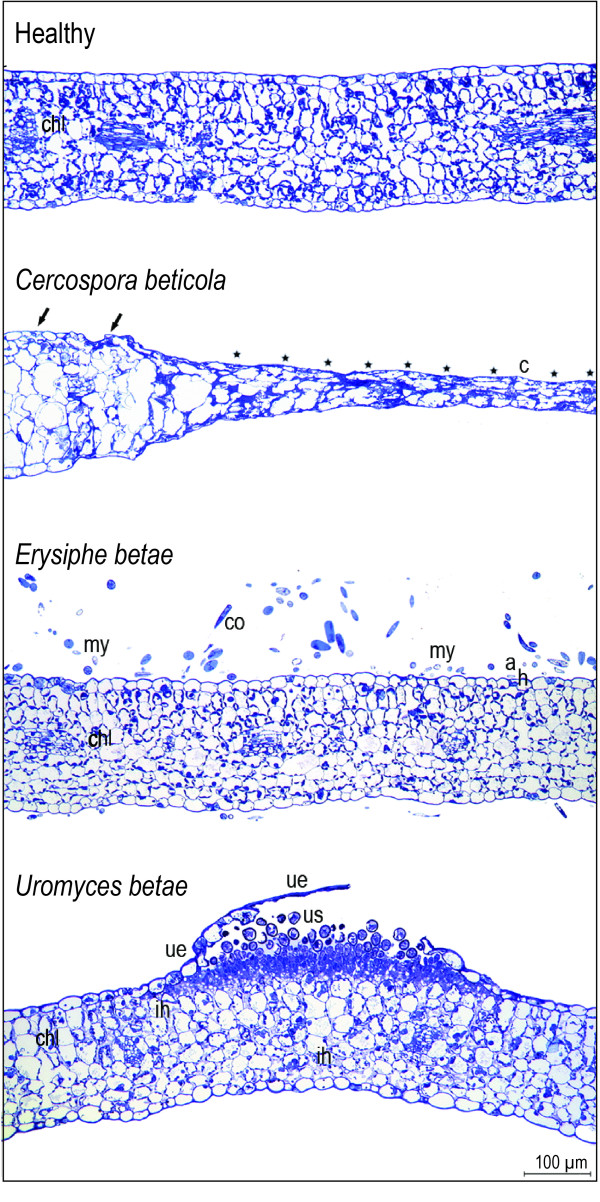
**Thin-sections of healthy and infected sugar beet leaves stained with toluidine blue: healthy leaf with intact leaf structure, chloroplasts (chl) are located peripherally in the cells**. Leaf infected with *Cercospora beticola*.at the border of tissue without macroscopic symptoms (marked with arrows) to symptomatic necrotic tissue (marked with asterisks) in the centre (c) of a mature symptom. *Erysiphe betae *infected leaf with mycelium (my) and conidia (co) on the upper leaf surface. The fungus has penetrated the epidermal cell wall, appressoria (a) and haustoria (h) were formed. Leaf with a ruptured pustule of *Uromyces betae*. The upper epidermis (ue) is detached from parenchyma, urediniospores (us) were released, and intercellular hyphae (ih) appear in the intercellular space of the mesophyll.

Light microscopy visualized the modifications in the tissue structure of sugar beet leaves resulting from the activities of the fungal pathogens *C. beticola, E. betae*, and *U. betae*; obvious differences to the morphology of healthy leaves occurred (Figure 2). *Cercospora beticola *penetrated the leaf through stomata. Intercellular hyphae were formed and pseudostromata were developed in the substomatal leaf tissue. At the edge between CLS lesions and healthy tissue, deep splits and sulcate leaf tissue occurred (Figure 2B). The CLS symptom obviously was subsided; cell lysis with minor intercellular space was accumulated in the necrotic centre of CLS symptoms.

Superficial mycelia and chains of conidia were characteristic symptoms of PM. Thin-sections of *E. betae *infected sugar beet leaves showed minor influence of the pathogen on tissue structure (Figure 2C). Mycelium and conidia were observed on the upper leaf surface and less frequently on the lower leaf surface. The superficially growing pathogen penetrates the epidermal cell wall after appressoria formation and produces haustoria within epidermal cells; the formation of a new haustorium requires another appressorium and the penetration of the epidermal layer.

In cross sections of SBR pustules produced by *Uromyces betae*, a swelling of leaf tissue, caused by initial spore accumulation under the epidermis, was observed (Figure 2D). In advanced stages of pathogenesis, accumulated urediniospores breached the epidermal layer. The roundish urediniospores were released and spread onto the neighbouring leaf area. Intercellular hyphae filling the intercellular space of the mesophyll were detected next to the pustule.

### Role of spatial resolution in the detection and identification of leaf diseases

**Figure 3:**
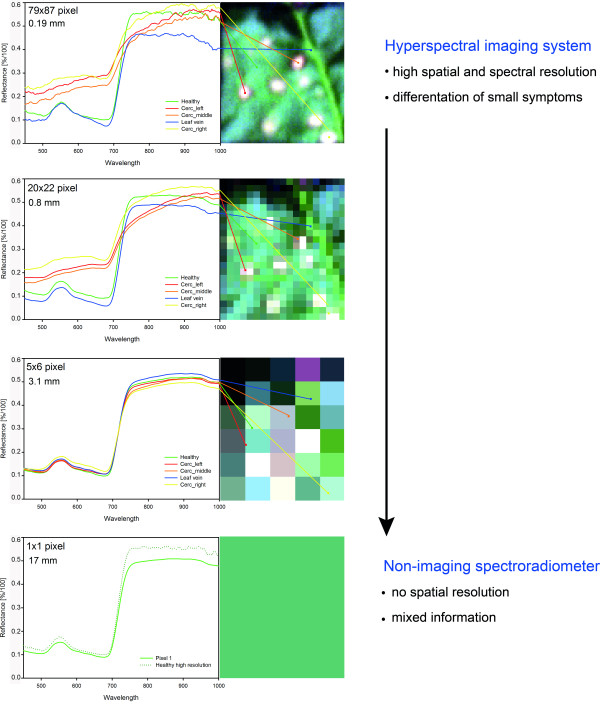
**Effect of spatial resolution of hyperspectral information on sensitivity of sensor for the detection of *Cercospora *leaf spot symptoms**. Cubic convolution was used for spectral resampling of the original spatial resolution of 0.19 mm (79 × 87 pixels covering an area of 289 mm^2^) to simulate resolutions of 0.8 mm (20 × 22 pixels), 3.1 mm (5 × 6 pixels) and 17 mm (1 pixel), respectively.

The spatial resolution of a sensor system is crucial for the detection and identification of leaf diseases. A spatial resolution of 0.2 mm per pixel was optimal to visualize characteristic leaf spots caused by *C. beticola *(Figure 3). With decreasing spatial resolution the amount of pixels with mixed information increased and the differences in reflectance decreased. At a spatial resolution of 3.1 mm, characteristic symptoms were not detectable anymore; the spectral signal was made up of both, healthy and diseased tissue. The signal from a spatial resolution of 17.1 mm was similar to that measured with a non-imaging spectroradiometer. Essential information was lost by averaging the reflectance of healthy and diseased tissue of the measuring area.

### Spectral signatures of characteristic regions of a mature symptom

**Figure 4:**
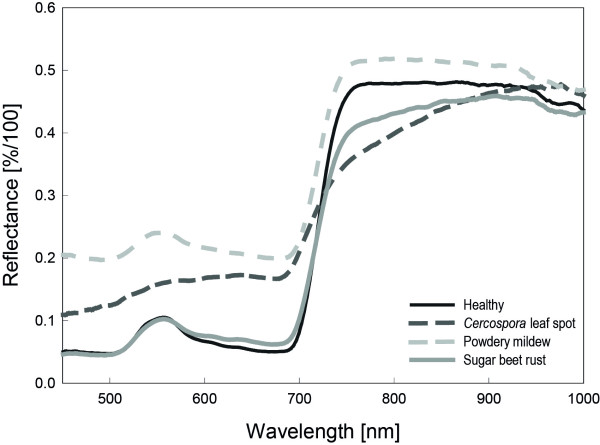
**Spectral signatures of healthy sugar beet leaves and from the centre of mature symptoms of sugar beet leaves diseased with *Cercospora *leaf spot, sugar beet rust and powdery mildew, respectively**. Reflectance spectra represent the mean of n = 20 pixel of characteristic tissue.

**Figure 5:**
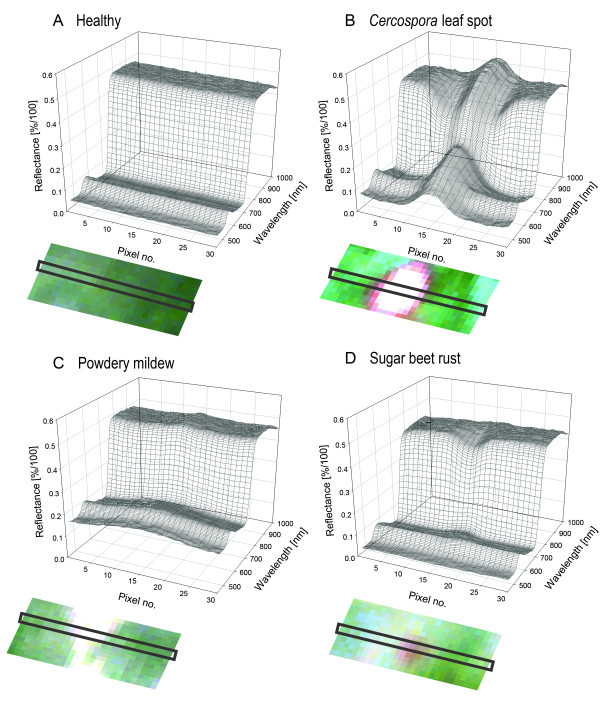
**Reflectance continuum from healthy tissue and disease specific symptoms**. Spectral signatures were extracted pixel-wise from a transect through characteristic leaf tissue from hyperspectral imaging. Reflectance of (A) healthy tissue and mature symptoms of (B) *Cercospora *leaf spot, (C) powdery mildew, and (D) sugar beet rust. Characteristic symptoms are shown as RGB images beneath their corresponding reflectance spectra. The pixels from which reflectance spectra were extracted are indicated with a black rectangle.

Spectral signatures from the centre of mature symptoms differed between the diseases (Figure 4). Reflectance of *Cercospora *leaf spot diseased pixel increased in the VIS and decreased in the NIR compared to healthy pixels. Powdery mildew causes and overall increase of reflectance, whereas sugar beet rust causes minor changes in the VIS from 550 to 700 nm and a decrease in the NIR. The spectral signatures from transects through healthy leaf tissue are plotted in Figure 5A; each spectrum belongs to a pixel of the transect. Spectral reflectance of healthy leaf tissue from adjacent pixels over a leaf segment was quite homogeneous. Minor variation was due to slight heterogeneity of the leaf tissue, the surface structure of sugar beet leaves and its interplay with irradiance. Spectral reflectance from transects through mature CLS symptoms showed obvious differences depending on the region of the symptom (Figure 5B). The margin of leaf spots had higher reflectance in the VIS and lower reflectance in the NIR. Spectra from the necrotic centre were characterized by increased reflectance in the VIS and the NIR region. Reflectance of leaf tissue covered by PM increased throughout the spectrum, depending on the density of the fungal mycelium on leaf surface (Figure 5C). Spectral reflectance of pixels from the margin of PM colonies was characterized by strong increase in the VIS and minor increase in the NIR. Denser mycelia in the centre of the colonies caused a more pronounced reflectance increase. Spatial changes in spectral signatures caused by SBR were less obvious, in fact of the small size of rust colonies and less destructive interactions with the host plant (Figure 5D). The transition area from healthy tissue to rust pustules was characterized by a general decrease in reflectance, whereas the centre of rust pustules had lower reflectance around the green peak (550 nm).

### Spatiotemporal dynamics of spectral patterns during pathogenesis

**Figure 6:**
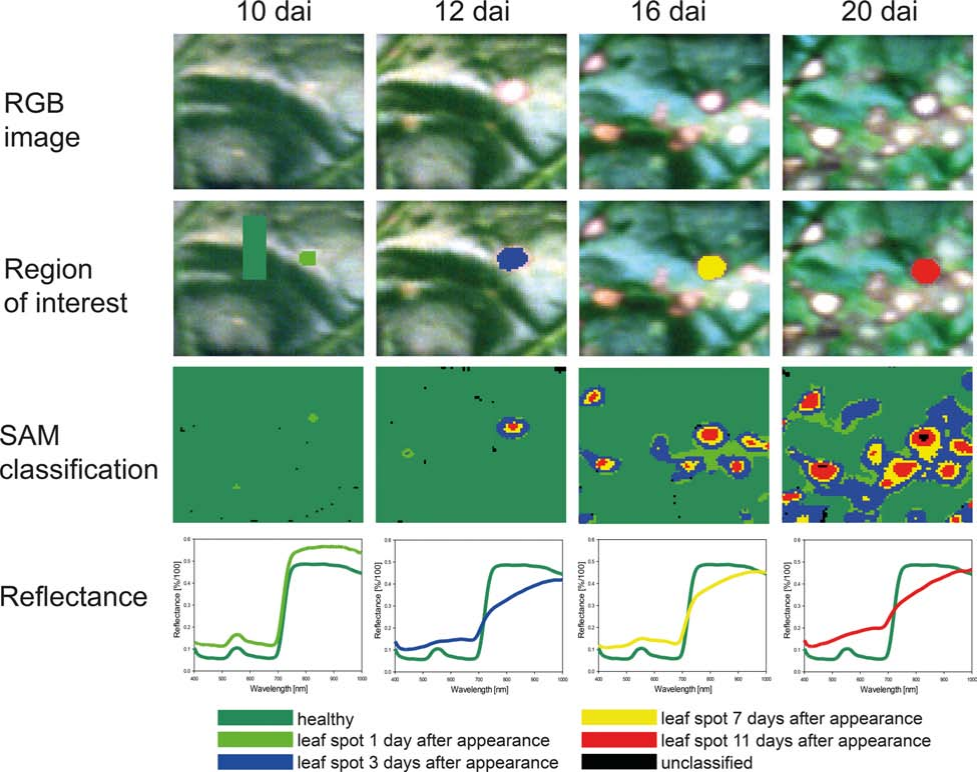
**Spatial and temporal dynamics in hyperspectral signature of *Cercospora *leaf spot during pathogenesis**. RGB images of infected sugar beet tissue (first row); region of interest (ROI) for the extraction of characteristic spectral signatures for the classes healthy tissue (light green), leaf spot 1 (light green) 3 (blue), 7 (yellow) and 11 (red) days after appearance, respectively (second row); Spectral Angle Mapper (SAM) classification based on spectral signatures from ROIs (third row); spectral signatures for each class (last row).

Since subareas of characteristic, mature symptoms of sugar beet leaf diseases varied in their spectral signature, changes of the spectral reflectance of infected leaf tissue in time and space was investigated during pathogenesis of the diseases. As exemplified for CLS in Figure 6, reflectance of infected leaf segments was analyzed at several times after appearance of first visible symptoms covering the time span of the development of typical disease symptoms. The process of symptom development has been monitored using mean reflectance of symptoms at different stages as endmembers in SAM classification. Spectral signature of CLS - tiny spots of discoloured tissue - one day after first appearance showed marginal differences to healthy leaf tissue. With further symptom development, this tissue reaction and spectral signature spread at the head of the growing leaf spot whereas the spectral signature and reaction of the primary infected tissue changed to dry, necrotic tissue (centres of leaf spots in Figure 6). Typical CLS symptoms 20 dai included almost concentric rings of tissue differing in spectral signature; the signatures turn from one to the other with time.

Similar spatiotemporal developments were detected also for PM and SBR. For PM, reflectance of diseased tissue in the VIS and NIR consistently increased. Reflectance of the centre of colonies three days after first appearance in the VIS was significantly higher than that of the margin of the colonies and of colonies one day after appearance.

### Classification and differentiation of symptoms and quantification of disease using the Spectral Angle Mapper

Based on hyperspectral imaging data the spectral angle classification algorithm was used for the identification of different subareas of disease-specific symptoms and their quantification (Tab. 1). Results of the SAM classification, summarized in Table 1 were validated using confusion matrices (provided as additional file 1, Table S1-S3). For CLS classification, the three classes 'healthy tissue', 'margin', and 'necrotic centre' of CLS were chosen. On the first day of the measuring period 99.89% of total leaf area was classified as healthy leaf tissue with an overall classification accuracy of 99.88%. The very high kappa coefficient underlines the agreement between ground truth and classification result. Eight days after inoculation, only 1.1% of healthy leaf tissue was unclassified with an overall accuracy of 98.90% (kappa coefficient 0.99). Classification accuracy decreased to 89.58% (kappa coefficient 0.52) 11 dai, because parts of the margin of CLS was falsely classified as healthy (11.18%). With higher disease severities and mature symptoms, classification accuracy increased again (96.58%, kappa coefficient 0.92, 14 dai). Seventeen days after inoculation, 79.05% of leaf area was quantified as healthy by the SAM, and 20.95% as CLS.

**Table 1:** Classification accuracy [%] of spectral angle mapper classification of *Cercospora *leaf spot and powdery mildew diseased leaves during disease progress and quantification of diseased leaf area by SAM classification.

Disease		Days after inoculation				
		8	11	14	17	20
*Cercospora *leaf spot	Overall accuracy [%]	98.90	89.01	96.58	98.73	
	
	Kappa coefficient	0.99	0.53	0.92	0.98	
	
	Diseased leaf area [%]	0	6.56	7.61	20.92	

Powdery mildew	Overall accuracy [%]	94.34	96.79	97.23	90.18	
	
	Kappa coefficient	0.88	0.91	0.95	0.84	
	
	Diseased leaf area [%]	14.40	19.37	41.94	50.04	

Sugar beet rust	Overall accuracy [%]					61.70
	
	Kappa coefficient					0.56
	
	Diseased leaf area [%]					2.89

**Figure 7:**
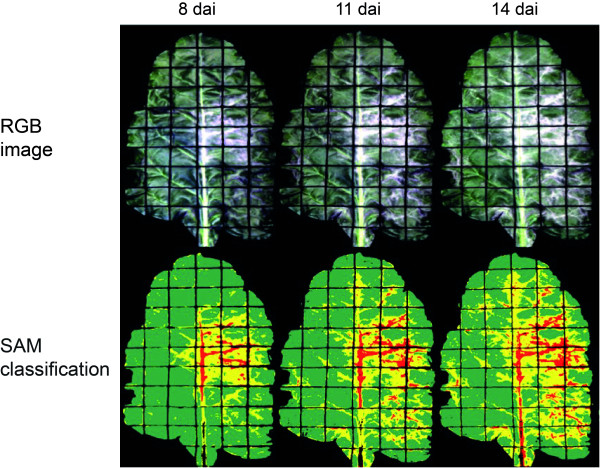
**Automatic classification of powdery mildew on sugar beet leaves using spectral angle mapper (SAM) algorithm**. The three classes 'healthy' (green), 'light mycelium' of powdery mildew (yellow), and 'dense mycelium' of powdery mildew (red) were separated at different disease severity stages with a maximum angle threshold of 0.1°. RGB images and false colour SAM classification images 8 dai, 11 dai, and 14 dai.

For the classification and quantification of powdery mildew, the three classes 'healthy tissue', 'light mycelium' and 'dense mycelium' were chosen (Figure 7, additional file 1, Table S2). Leaves were classified healthy 100% in hyperspectral images taken just before inoculation (0 dai); classification accuracy was 100% (kappa coefficient = 1.00; Tab. 1). Visible powdery mildew colonies appeared 8 dai in the right middle of the sugar beet leaf and next to the branching leaf veins (Figure 7). Overall classification accuracy was 94.34% (kappa coefficient 0.88) at this time. During the time of symptom appearance and colony growth the results of SAM classification coincided with visually assessed disease symptoms (Figure 7). The SAM gave high classification accuracies 11, 14 and 17 dai, respectively (Tab. 1). The differentiation between light and dense mycelium of powdery mildew, however, proved to be difficult.

Due to the small size of SBR symptoms and the low disease severity during the measuring period, classification of SBR by the SAM algorithm was difficult (additional file 1, Table S3). First rust pustules became visible 20 days after inoculation. Before this day, no classification of SBR inoculated sugar beet leaves was possible (Tab. 1). The post classification based on ground truth data yielded in an overall classification accuracy of 61.70% with a low kappa coefficient of 0.56 with 15.98% unclassified healthy leaf area and 15.12% unclassified symptoms of SBR. Satisfying differentiation between healthy leaf parts and symptoms of SBR were also not feasible. An amount of 20.67% of the rust pustules was classified as healthy and vice versa, 4% of the healthy tissue was classified as SBR.

## Discussion

Hyperspectral imaging proved to be highly suitable for the detection, identification and quantification of fungal diseases on the leaf level. Each disease influences the spectral reflectance of sugar beet tissue in a specific way resulting in disease-specific spectral signatures. Similar effects have been described previously for foliar and soil-borne diseases of sugar beet by Mahlein *et al. *[5] and Hillnhütter *et al. *[22] using non-imaging hyperspectrometry.

Since the portion of a signal from diseased tissue in a mixed signal depends on disease severity, the sensitivity and specificity of non-imaging spectroradiometers is limited. Especially at low disease severities, spectra are based on high percentage of reflectance from healthy tissue and only a low portion of symptomatic tissue causing changes in the spectrum. Diseased plants or leaves may be detected by using non-imaging spectrometry, however, only imaging techniques with high spatial resolution - i.e. operating in proximity to the objects - allow for the detection, identification, and quantification of disease symptoms. Disadvantages of non-imaging spectroradiometer, including the separation of mixed infection with two or more diseases on the same leaf or plant, can be overcome by the use of HSI technology.

A pixel-wise attribution of disease-specific symptoms and healthy tissue is conducive to observe spectral reflectance patterns of foliar diseases in detail. Some disease symptoms can only be distinguished from other diseases and stresses when hyperspectral imaging with high spatial resolution is used [17,14]. The detection limit of a non-imaging spectroradiometer was 10% diseased leaf area for CLS and powdery mildew and 20% for SBR, respectively [5]. In contrast, single symptoms can be detected and identified by using HSI systems, since pure signals from pixels of diseased tissue are recorded.

High spatial resolution is crucial in particular for the detection of leaf diseases with discrete, roundish symptoms like CLS or SBR. Spatial resolution of the hyperspectral camera used in this study provided information even on subareas of disease symptoms. Nevertheless, the tiny uredinia of *U. betae *and limited spatial resolution of the sensor resulted in a high amount of mixed pixels in SBR experiments. Depending on the shape of the symptoms, pixel size should be smaller than the object of interest by a factor of 2 to 5 [23,24]. This rule from remote sensing restricts the sensing of plant diseases to proximal sensing technologies.

The sugar beet diseases differed in their temporal and spatial development as well as in their effects on plant tissue associated to reflectance characteristics. The spectral impact of sugar beet diseases on leaf reflectance was previously described in detail by Mahlein et al. [5] On hyperspectral imaging cubes along transects through CLS symptoms revealed a continuum from healthy tissue, over newly colonized tissue, discoloured (reddish-brown) tissue, chlorotic cells, dying cells and dead cells in the centre of mature associated with characteristic changes of reflectance in the VIS and NIR, which is especially sensitive to modifications of the tissue structure [25,26]. Boyer *et al. *[27] described similar effects in senescent leaves of the northern pine oak.

The biotrophic pathogens *U. betae *and *E. betae *are less destructive; both pathogens largely rely on the integrity of host cells and functionality of metabolism of their host plant. Structural changes of infected leaf tissue and modifications in pigment content were smaller than for CLS-diseased leaves and resulted in only slight changes in VIS and NIR reflectance. The number of chloroplasts was not visually affected at the time of the appearance of mature symptoms. The whitish mycelium of *E. betae *on the surface increased tissue reflectance over the full range of the sensor. An unambiguous detection of powdery mildew in early stages is challenging since the dust-like cover results in a parallel shift of reflectance with minor influence on the shape of the reflectance curve. The reddish-brown urediniospores of *Uromyces betae*, in contrast, influenced tissue reflectance similar to the reddish-brown margin of CLS symptoms (minor decrease from 450 to 500 nm, increase from 550 to 700 nm, decrease in NIR, Figure 4). High concentrations of carotenoids and melanin-like pigments, causing the characteristic brown-orange colour of urediniospores are well documented for many rust fungi [28]. As stated by Gitelson et al. [29] carotenoids and chlorophyll have overlapping absorption bands in the blue range around 520 nm. *Cercospora *leaf spots cause an increase in reflectance from 400 to 550 nm whereas sugar beet rust causes no increase in reflectance in this range. Reflectance is constant with roundabout 0.05%/100; it is assumed that the carotenoids described for sugar beet rust urediniospores counteract the effect of the chlorophyll loss. Nevertheless, the small size of SBR colonies impeded the detection in early stages or at low disease severity.

Spatial patterns of discrete symptoms of sugar beet diseases could be investigated by pixel-wise assignment of spectral signatures. Modifications of spectral reflectance at different developmental stages were displayed in spectral signatures of different subareas of the symptoms. For instance reflectance of new, immature symptoms was similar to that from the margin of fully developed lesions. The results for powdery mildew and SBR generally confirm the principle that maturing, but still growing disease symptoms include all developmental stages so far.

Specific effects of diseases, disease stage, and the impact of disease severity on spectral characteristics of plants are complex, but may allow for new insights into host-pathogen interactions [30]. Similar to Ustin and Gamon [31], who classified different plant functional types based on morphological and physiological traits into 'optical types' by reflectance measurements, spectra of subareas of infected tissue categorised during disease development in a similar way. Hyperspectral imaging clarified various stages of sugar beet diseases as a continuum rather than discrete classes. Gradients of reflectance exist between healthy/asymptomatic and symptomatic tissue which may impede the classification between healthy and diseased leaf areas. The development of patterns in time and space, recorded by hyperspectral imaging may help to identify disease or stress influencing crops on the canopy level [32] and on the tissue level [30].

Given that the spectral patterns of healthy and diseased tissue are known, supervised classification was the choice to detect, identify, and quantify diseased tissue of sugar beet leaves. Since SAM classification is based on defined endmember spectra, the detection of leaf colonization prior to the occurrence of visible symptoms was not feasible by following this approach, but visible symptoms were classified with high accuracy. Benefits of the SAM algorithm for disease detection are insensitivity to heterogeneities of surface topography and illumination, because the angle between two vectors is invariant with respect to the length of the vectors [33]. Leaf veins and differences in growth rates cause a characteristic undulated, grooved topography of sugar beet leaves depending on the genotype. Heterogeneities in reflectance intensity occur, as radiation is not reflected straightforward by these surfaces. Although classification accuracy of SAM was satisfying, it should be mentioned that this classification algorithm uses the average spectrum of each endmember class (e.g. healthy and different symptom peculiarities). The spectral variability within each endmember class, denoted as intra-class variability is not retained. Luc *et al. *[34] obtained a higher overall classification accuracy of Belgian coastline regions by modifying the common SAM to an optimized SAM preserving the intra-class variability. This approach may also resolve problems in disease classification, e.g. lower accuracy for early disease stages when only immature symptoms occur. Similar to the problems in the tomato - *P. infestans *system described by Zhang *et al. *[35] low disease levels of SBR resulted in lower accuracy of the SAM algorithm in this study.

## Conclusions

This study is the first analysis of characteristic symptoms of various sugar beet diseases and their development in time and space using HSI. This kind of pathogen 'life-logging' with HSI is of high interest for various applications from basic research on the cellular level to large scale applications in agricultural fields. New insights from hyperspectral disease detection on sugar beet make a contribution to a better understanding of plant optical properties during pathogenesis. Different analysis methods and sensor specificities can be transferred and generalized for other plant-pathogen systems. The technology enables the development of precise high-throughput screening systems of plant diseases for resistance breeding and fungicide development.

## Materials and Methods

### Plant material

Sugar beet seeds (*Beta vulgaris *L., cv. Pauletta, KWS, Einbeck, Germany) were pre-grown in small pots and were piqued when the primary leaves had fully developed. Seedlings were transferred into a commercial substrate (Einheitserde Topf, Klasmann-Deilmann, Geeste, Germany) in plastic pots (Ø 170 mm) at 23/20°C (day/night), 60% relative humidity (RH) and a photoperiod of 16 h. Plants were watered daily and fertilized weekly with 100 ml of a 0.2% solution of Poly Crescal (Aglukon, Düsseldorf, Germany) and were used for the experiments after reaching growth stage (GS) 16 [36].

### Culture and inoculation of pathogens

Conidia of *C. beticola *were harvested from diseased sugar beet leaves, sampled from fields in autumn and incubated in a moist chamber for 12 h. *Cercospora beticola *was inoculated by spraying a spore suspension (4 × 10^4 ^conidia ml^-1^) onto leaves using a hand sprayer. Subsequently, plants were covered with plastic bags to realize 100% RH at 25/20°C for 48 h.

Urediniospores of *U. betae *were brushed off diseased leaves and were stored at -19°C. Suspensions of *U. betae *(4 x10^4 ^urediniospores ml^-1^) were sprayed onto leaves before covering sugar beets with plastic bags and incubating them for 48 h at 19/16°C. For further incubation the plants inoculated with *C. beticola *and *U. betae *were transferred to the greenhouse at 23/20°C and 60 ± 10% RH.

Plants heavily infested with PM were used as inoculum source of *E. betae*. Healthy plants were inoculated in a chamber where a ventilator ran for 25 seconds in order to distribute *E. betae *conidia evenly on the leaves. Plants were left over night and afterwards transferred to the greenhouse. Non-inoculated plants were kept as healthy controls at 23/20°C and 60 ± 10% RH in the greenhouse.

### Technical setup and hyperspectral image acquisition

For image acquisition sugar beet plants were placed on mobile tables (0.8 m × 0.8 m, four plants per table) 2 days after inoculation (dai). According to Chaerle *et al. *[37] the fifth fully developed leaf pair of each sugar beet plant was fixed horizontally on a frame between a grid pattern made of two layers of rubber-laminated mesh wire. Frame and grid pattern were coated with black, matte colour to reduce reflectance of the material. The mesh wire largely avoided movements of leaves which were subdivided into equally-sized squares (20 × 20 mm) on the images.

The hyperspectral imaging system combines an imaging spectrograph and a mirror scanner. The line scanning spectrograph ImSpector V10E (Spectral Imaging Ltd., Oulu, Finland) has a spectral range from 400 to 1000 nm and a spectral resolution of up to 2.8 nm. The maximal image size of the 30 μm sensor slot results in 1600 pixels per line with a sensor pixel size of 0.0074 mm. Limited by the distance between target and sensor system (0.60 m) a spatial resolution of 0.19 mm per pixel was obtained. A mirror scanner (Spectral Imaging Ltd.) - maximal field of view 80° - mounted in front of the objective lens provided the second spatial dimension of the images. The hyperspectral sensor system was mounted on a manual positioning XY-frame, surrounded by six ASD-Pro-Lamps (Analytical Spectral Devices Inc., Boulder, USA) radiating a near-solar light spectrum. The distance between lamps and leaves was 0.5 m with a vertical orientation of 45°. Imaging data were recorded in a dark chamber in order to realize optimal and reproducible illumination and constant measurement conditions. Hyperspectral images were taken daily from 2 dai until 21 dai.

Using the software SpectralCube (Spectral Imaging Ltd., Oulu, Finland) the angle of the mirror scanner as well as the spectral and spatial resolution were adapted to the object. Images on leaf level were taken with spectral binning 4 and spatial binning 1. Frame rate and exposure time were adjusted to binning and object. The sensor system was focused manually to a barium sulphate calibration bar (Spectral Imaging Ltd., Oulu, Finland) with black rhombi on a white background, placed in the same distance to the camera as the leaves. For subsequent calculation of reflectance, three images were grabbed. A dark current image was recorded by closing an internal shutter of the camera, followed by an image of a white reference bar (Spectral Imaging Ltd., Oulu, Finland), with the same horizontal size and on the same level as the object area, both with the same exposure time. Subsequently an image of the leaf area was recorded with improved exposure time. Experiments were conducted at least twice.

### Normalization and pre-processing of hyperspectral images

Calculations of reflectance, relative to a white reference bar and the dark current measurement were performed using the software ENVI 4.6 + IDL 7.0 (ITT Visual Information Solutions, Boulder, USA). After this normalization the Savitzky-Golay filter [38] was applied to smooth the signals from hyperspectral images. The parameters for the smoothing process were 5 supporting points to the left and right, respectively, and a fifth degree polynomial. The pre-processed images were used for further analysis using ENVI 4.6 + IDL 7.0.

### Reduction of spatial resolution by cubic convolution

The resampling function 'cubic convolution' was applied on hyperspectral images to reduce the spatial resolution of the original data. Calculation of new pixel values was performed by weighing 16 surrounding pixels. From a primary spatial resolution of 0.19 mm pixel size, reduced spatial resolutions of 0.8 mm, 3.1 mm and 17 mm pixel size were calculated.

### Disease-specific spectral signatures

Spectral signatures of pixels from characteristic regions of fully developed disease symptoms were extracted. Twenty fully developed symptoms of the same developmental stage were analysed for each disease. As the characteristics of symptoms vary during pathogenesis, spectral signatures of symptoms at different stages were collected and averaged. Ten symptomatic areas were analysed daily for each disease. Spectral signatures from infested areas were extracted from regions of interest (ROIs). Additionally RGB images of sugar beet leaves were taken and the leaf infection of each pathogen was evaluated visually and classified as percentage indicating the fraction corresponding to disease area.

### Spectral Angle Mapper (SAM) classification

Automatic classification known from remote sensing image analysis was applied to hyperspectral images of diseased sugar beet leaves for the differentiation of diseases. The Spectral Angle Mapping method (SAM, [33]) was performed using the software ENVI 4.6 + IDL 7.0. Spectral classification approaches assign each pixel to one out of several known categories or classes (endmembers) through a statistical approach. Spectrally unique signatures of pure image components, i.e. endmembers, have to be defined, and specific classification algorithms can be calculated to classify the pixel. For CLS classification the endmembers 'healthy', 'margin' of a leaf spot, and 'centre' of a leaf spot were chosen, for PM 'healthy', 'light mycelium' and 'dense mycelium', and for classification of SBR 'healthy' and 'rust'. The data set was divided into a set of training data and a set of test data, to train the classifiers. The classification decomposes the hyperspectral image into a false colour image, containing thematic information of the previously selected classes. SAM calculates the spectral similarity of spectra and reference spectra using the spectral angle between the two spectra in an n-dimensional space dependent on the number of spectral bands. The output of SAM is an angular difference for each pixel, which can be illustrated in a false colour image; small spectral angles correspond to high similarity, large spectral angles to low similarity [33]. Because the analysed spectra are transferred as vectors, variable illuminations due to the surface structure and veins of sugar beet leaves were attenuated (darker pixel will plot along the same vector, but closer to the origin). The SAM result was validated by the overall accuracy, quantifying the percentage of cases correctly classified and the kappa coefficient which accommodates for the effects of change agreement.

### Microscopic investigations

Sugar beet leaf tissue from non-infected leaves and leaves infected with the pathogens (3 - 5 mm × 3 - 5 mm) was sampled for histological analysis. Specimens were fixed with 8% paraformaldehyde and 8% glutaraldehyde in 0.2 M sodium cacodylate buffer (pH 7.3) under vacuum for 4 h at room temperature [39]. Samples were washed three times in cacodylate buffer for 20 min each, dehydrated in a graded ethanol series, and embedded in London Resin white medium. The embedded tissue was semi-thin sectioned with a diamond knife on an ultra-microtome (Reichert Ultracut E; Leica Microsystems, Nussloch, Germany) and was stained in 1% toluidine blue. Stained samples were observed with a Leitz DMR 6000B photomicroscope. Digital photos were taken using a digital camera (JVC, Ky-F75U) and the software Discus, 4.6 (Technical Office Hilgers, Königswinter, Germany).

## List of abbreviations

CLS: *Cercospora *leaf spot; GS: Growth stage; HSI: Hyperspectral imaging; NIR: Near infrared; PM: Powdery mildew; RH: Relative humidity; ROI: Region of interest; SAM: Spectral angle mapper; SBR: Sugar beet rust; VIS: Visible range.

## Competing interests

The authors declare that they have no competing interests.

## Authors' contributions

AKM, US and ECO designed the study, interpreted the data and drafted the manuscript. AKM and CH developed the measuring procedure for the assessment and pre-processing of hyperspectral images. AKM carried out the hyperspectral measurements and the statistical analysis. All authors read and approved the final manuscript.

## Supplementary Material

Additional file 1 **Confusion matrix for SAM classification of *Cercospora *leaf spot, powdery mildew and sugar beet rust diseased leaves. Classification accuracy [%] and Kappa coefficient during disease progress and quantification of diseased leaf area by SAM classification**. Confusion matrixes (Table S1-S3) for the SAM classification of *Cercospora *leaf spot, powdery mildew and sugar beet rust diseased leaves for each measuring date. Click here for file

## References

[B1] Blackburn GA Hyperspectral remote sensing of plant pigments J Exp Bot 2007 58 844 867 10.1093/jxb/erl123 16990372

[B2] Gitelson AA Gritz Y Merzylak MN Relationships between leaf chlorophyll content and spectral reflectance and algorithms for non-destructive chlorophyll assessment in higher plant leaves J Plant Physiol 2003 160 271 282 10.1078/0176-1617-00887 12749084

[B3] Jacquemoud S Ustin SL Leaf optical properties: A state of the art Proceedings 8th International Symposium Physical Measurements & Signatures in Remote Sensing 2001 CNES, Aussois (France) 223 232

[B4] Delalieux S van Aardt J Keulemans W Coppin P Detection of biotic stress (*Venturia inaequalis*) in apple trees using hyperspectral data: Non-parametric statistical approaches and physiological implications Eur J Agron 2007 27 130 143 10.1016/j.eja.2007.02.005

[B5] Mahlein AK Steiner U Dehne HW Oerke EC Spectral signatures of sugar beet leaves for the detection and differentiation of diseases Precis Agric 2010 11 413 431 10.1007/s11119-010-9180-7

[B6] Rumpf T Mahlein AK Steiner U Oerke EC Dehne HW Plümer L Early detection and classification of plant diseases with Support Vector Machines based on hyperspectral reflectance Comput Electron Agr 2010 74 91 99 10.1016/j.compag.2010.06.009

[B7] Steddom K Bredehoeft MW Khan M Rush CM Comparison of visual and multispectral radiometric disease evaluations of Cercospora leaf spot of sugar beet Plant Dis 2005 89 153 158 10.1094/PD-89-0153 30795217

[B8] Oerke EC Steiner U Dehne HW Lindenthal M Thermal imaging of cucumber leaves affected by downy mildew and environmental conditions J Exp Bot 2006 57 2121 2132 10.1093/jxb/erj170 16714311

[B9] Gamon JA Surfus JS Assessing leaf pigment content and activity with a reflectometer New Phytol 1999 143 105 117 10.1046/j.1469-8137.1999.00424.x

[B10] Pinter PJ Hatfield JL Schepers JS Barnes EM Moran MS Daugthry CST Upchurch DR Remote sensing for crop management Photogramm Eng Rem Sens 2003 69 647 664

[B11] Moran MS Inoue Y Barnes EM Opportunities and limitations for image-based remote sensing in precision crop management Remote Sens Environ 1997 61 319 346 10.1016/S0034-4257(97)00045-X

[B12] Carter GA Knapp AK Leaf optical properties in higher plants: linking spectral characteristics to stress and chlorophyll concentration Am J Bot 2001 88 677 684 10.2307/2657068 11302854

[B13] Steiner U Bürling K Oerke EC Sensorik für einen präzisierten Pflanzenschutz Gesunde Pflanz 2008 60 131 141 10.1007/s10343-008-0194-2

[B14] West JS Bravo C Oberti R Moshou D Ramon H McCartner HA Oerke EC, Gerhards R, Menz G, Sikora RA Detection of fungal diseases optically and pathogen inoculums by air sampling Precision Crop Protection - the Challenge and Use of Heterogeneity 2010 Springer, Dordrecht, Netherlands 135 149

[B15] Bock CH Poole GH Parker PE Gottwald TR Plant disease severity estimated visually, by digital photography and image analysis, and by hyperspectral imaging Crit Rev Plant Sci 2010 29 59 107 10.1080/07352681003617285

[B16] Chaerle L Van der Staeten D Imaging techniques and the early detection of plant stress Trends Plant Sci 2000 5 495 501 10.1016/S1360-1385(00)01781-7 11077259

[B17] Bravo C Moshou D West J McCartney A Ramon H Early disease detection in wheat fields using spectral reflectance Biosyst Eng 2003 84 137 145 10.1016/S1537-5110(02)00269-6

[B18] Nansen C Tulio M Swanson R Weaver DK Use of spatial structure analysis of hyperspectral data cubes for detection of insect-induced stress in wheat plants Int J Remote Sens 2009 30 2447 2464 10.1080/01431160802552710

[B19] Polder G van der Heijden GWAM van Doorn J Clevers JGPW van der Schoor R Baltissen AHMC Detection of the tulip breaking virus (TBV) in tulips using optical sensors Precis Agric 2010 11 397 412 10.1007/s11119-010-9169-2

[B20] Balasundaram D Burks TF Bulanon DM Schubert T Lee WS Spectral reflectance characteristics of citrus canker and other peel conditions of grapefruit Postharvest Biol Tec 2009 51 220 226 10.1016/j.postharvbio.2008.07.014

[B21] Qin J Burks TF Ritenour MA Bonn WG Detection of citrus canker using hyperspectral reflectance imaging with spectral information divergence J Food Eng 2009 93 183 191 10.1016/j.jfoodeng.2009.01.014

[B22] Hillnhütter C Mahlein A-K Sikora RA Oerke E-C Remote sensing to detect plant stress induced by *Heterodera schachtii *and *Rhizoctonia solani *in sugar beet fields Field Crop Res 2011 122 70 77 10.1016/j.fcr.2011.02.007

[B23] Jensen JR Solid and hazardous waste disposal site selection using digital geographic information system techniques Sci Total Environ 1986 56 265 276

[B24] O'Neill RV Hunsaker CT Timmins SP Jackson BL Jones KB Ritters KH Wickham JD Scale problems in reporting landscape pattern at the regional scale Landscape Ecol 1996 11 169 180 10.1007/BF02447515

[B25] Buschmann C Nagel E In vivo spectroscopy and internal optics of leaves as basis for remote sensing of vegetation Int J Remote Sens 1993 14 711 722 10.1080/01431169308904370

[B26] Gates DM Keegan HJ Schelter JC Weidner VR Spectral properties of plants Appl Optics 1965 4 11 20 10.1364/AO.4.000011

[B27] Boyer M Miller J Belanger M Hare E Wu J Senescence and spectral reflectance in leaves of northern pin oak (*Quercus palustris *Muenchh.) Remote Sens Environ 1988 25 71 87 10.1016/0034-4257(88)90042-9

[B28] Davoli P Weber RWS Identification and quantification of carotenoid pigments in aeciospores of the daisy rust fungus, *Puccinia distincta* Phytochemistry 2002 60 309 313 10.1016/S0031-9422(02)00120-6 12031451

[B29] Gitelson AA Zur Y Chivkunova OB Merzlyak MN Assessing carotenoid content in plant leaves with reflectance spectroscopy Photochem Photobiol 2002 75 272 281 10.1562/0031-8655(2002)075<0272:ACCIPL>2.0.CO;2 11950093

[B30] Mahlein A-K Oerke EC Steiner U Dehne H-W Recent advances in sensing plant diseases for precision crop protection Eur J Plant Pathol 2011 DOI 10.1007/s10658-011-9878-z

[B31] Ustin SL Gamon JA Remote sensing of plant functional types New Phytol 2010 186 795 816 10.1111/j.1469-8137.2010.03284.x 20569415

[B32] Nutter F van Rij N Eggenberger SK Holah N Oerke EC, Gerhards R, Menz G, Sikora RA Spatial and temporal dynamics of plant pathogens Precision Crop Protection - the Challenge and Use of Heterogeneity 2010 Springer, Dordrecht, Netherlands 27 50

[B33] Kruse FA Lefkoff AB Boardman JW Heidebrech KB Shapiro AT Barloon PJ Goetz AFH The spectral image-processing system (Sips) - interactive visualization and analysis of imaging spectrometer data Remote Sens Environ 1993 44 145 163 10.1016/0034-4257(93)90013-N

[B34] Luc B Deronde B Kempeneers P Debruyn W Provoost S Liang S Optimized spectral angle mapper classification of spatially heterogeneous dynamic dune vegetation, a case study along the Belgian coastline9th International Symposium on Physical Measurements and Signatures in Remote Sensing (ISPMSRS). Beijing, China 2005

[B35] Zhang M Qin Z Liu X Ustin S Detection of stress in tomatoes induced by late blight disease in California, USA, using hyperspectral remote sensing Int J Appl Earth Obs Geoinf 2003 4 295 310 10.1016/S0303-2434(03)00008-4

[B36] Meier U Bachmann L Buhtz H Hack H Klose R Märländer B Weber E Phenological growth stages of sugar beet (*Beta vulgaris *L. ssp.) Codification and description according to the general BBCH scale (with figures) Nachrichtenbl Deut Pflanzenschutzd 1993 45 37 41

[B37] Chaerle L Hagenbeck D De Bruyne E Van Der Straeten D Chlorophyll fluorescence imaging for disease-resistance screening of sugar beet Plant Cell Tiss Org 2007 91 97 106 10.1007/s11240-007-9282-8

[B38] Savitzky A Golay JME Smoothing and differentiation of data by simplified least squares procedures Anal Chem 1964 36 1627 1639 10.1021/ac60214a047

[B39] Karnovsky E A formaldehyde glutaraldehyde fixative of osmolality for use in electron microscopy J Cell Biol 1965 27 137 138

