# Supplementary material for: Hyperspectral imaging for small-scale analysis of symptoms caused by different sugar beet diseases
Source: Plant Methods. 2012 Jan 24;8:3. doi: 10.1186/1746-4811-8-3 (PMC3274483; doi:10.1186/1746-4811-8-3)
Supplement: Additional file 1 — Confusion matrix for SAM classification of Cercospora leaf spot, powdery mildew and sugar beet rust diseased leaves. Classification accuracy [%] and Kappa coefficient during disease progress and quantification of diseased leaf area by SAM classification. Confusion matrixes (Table S1-S3) for the SAM classification of Cercospora leaf spot, powdery mildew and sugar beet rust diseased leaves for each measuring date. [file 1746-4811-8-3-S1.DOCX]

**Additional files**

**Additional file 1**

**Title:** Confusion matrix for SAM classification of *Cercospora* leaf spot, powdery mildew and sugar beet rust diseased leaves. Classification accuracy [%] and Kappa coefficient during disease progress and quantification of diseased leaf area by SAM classification.

**Description:** Confusion matrixes (Table 1-3) for the SAM classification of *Cercospora* leaf spot, powdery mildew and sugar beet rust diseased leaves for each measuring date.

**Table S1** Confusion matrix for SAM classification of *Cercospora* leaf spot diseased leaves for the classes Healthy, Margin, and Centre. Classification accuracy [%] and Kappa coefficient during disease progress and quantification of diseased leaf area by SAM classification.

| **Days after inoculation 8** | | | | |
| --- | --- | --- | --- | --- |
| *Class* | *Ground truth* | | | |
|  | Healthy | Margin | Centre | Total |
| unclassified | 1.1 | 0 | 0 | 1.1 |
| Healthy | 98.9 | 0 | 0 | 98.9 |
| Margin | 0 | 0 | 0 | 0 |
| Centre | 0 | 0 | 0 | 0 |
|  | ***Overall = 98.90*** | | ***Kappa = 0.99*** | |
| **Days after inoculation 11** | | | | |
| *Class* | *Ground truth* | | | |
|  | Healthy | Margin | Centre | Total |
| Unclassified | 0 | 6.67 | 0 | 0.53 |
| Healthy | 88.82 | 2.22 | 0 | 92.91 |
| Margin | 11.18 | 91.11 | 0 | 6.56 |
| Centre | 0 | 0 | 0 | 0 |
|  | ***Overall = 89.01*** | | ***Kappa = 0.53*** | |
| **Days after inoculation 14** | | | | |
| *Class* | *Ground truth* | | | |
|  | Healthy | Margin | Centre | Total |
| Unclassified | 0 | 0 | 0.1 | 0 |
| Healthy | 100 | 4.78 | 0 | 92.39 |
| Margin | 0 | 93.53 | 16.07 | 6.18 |
| Centre | 0 | 1.79 | 83.82 | 1.43 |
|  | ***Overall = 96.58*** | | ***Kappa = 0.92*** | |
| **Days after inoculation 17** | | | | |
| *Class* | *Ground truth* | | | |
|  | Healthy | Margin | Centre | Total |
| Unclassified | 0 | 0 | 0 | 0 |
| Healthy | 87.5 | 1.08 | 0 | 79.05 |
| Margin | 12.5 | 98.92 | 0 | 15.01 |
| Centre | 0 | 0 | 100 | 5.94 |
|  | ***Overall = 98.73*** | | ***Kappa = 0.98*** | |

**Table S2** Confusion matrix for SAM classification of powdery mildew diseased leaves for the classes Healthy, Light mycelium, and Dense mycelium. Classification accuracy [%] and Kappa coefficient during disease progress and quantification of diseased leaf area by SAM classification.

| **Days after inoculation 8** | | | | |
| --- | --- | --- | --- | --- |
| *Class* | *Ground truth* | | | |
|  | Healthy | Light mycelium | Dense mycelium | Total |
| unclassified | 0 | 0 | 3.49 | 0.41 |
| Healthy | 94.04 | 4.44 | 0 | 85.16 |
| Light mycelium | 5.96 | 94.07 | 0 | 9.69 |
| Dense mycelium | 0 | 1.48 | 96.51 | 4.74 |
|  | ***Overall = 94.34*** | | ***Kappa = 0.88*** | |
| **Days after inoculation 11** | | | | |
| *Class* | *Ground truth* | | | |
|  | Healthy | Light mycelium | Dense mycelium | Total |
| unclassified | 0 | 0 | 0 | 0 |
| Healthy | 99.92 | 12.62 | 0 | 80.63 |
| Light mycelium | 0.08 | 77.10 | 0 | 10.65 |
| Dense mycelium | 0 | 10.28 | 100 | 8.72 |
|  | ***Overall = 96.79*** | | ***Kappa = 0.91*** | |
| **Days after inoculation 14** | | | | |
| *Class* | *Ground truth* | | | |
|  | Healthy | Light mycelium | Dense mycelium | Total |
| unclassified | 0 | 0 | 0 | 0 |
| Healthy | 100 | 0 | 0 | 58.06 |
| Light mycelium | 0 | 93.63 | 6.82 | 20.38 |
| Dense mycelium | 0 | 6.37 | 93.18 | 21.56 |
|  | ***Overall = 97.23*** | | ***Kappa = 0.95*** | |
| **Days after inoculation 17** | | | | |
| *Class* | *Ground truth* | | | |
|  | Healthy | Light mycelium | Dense mycelium | Total |
| unclassified | 0 | 0 | 0.29 | 0.09 |
| Healthy | 100 | 0 | 0 | 49.87 |
| Light mycelium | 0 | 88.07 | 25 | 25.71 |
| Dense mycelium | 0 | 11.93 | 74.71 | 24.33 |
|  | ***Overall = 90.18*** | | ***Kappa = 0.84*** | |

**Table S3** Confusion matrix for SAM classification of sugar beet rust diseased leaves for the classes Healthy and Dense mycelium. Classification accuracy [%] and Kappa coefficient during disease progress and quantification of diseased leaf area by SAM classification.

| **Days after inoculation 20** | | | |
| --- | --- | --- | --- |
| *Class* | *Ground truth* | | |
|  | Healthy | Rust | Total |
| unclassified | 15.98 | 15.12 | 15.98 |
| Healthy | 80.02 | 20.67 | 82.13 |
| Sugar beet rust | 4.00 | 64.21 | 2.89 |
|  | ***Overall = 61.70*** | | ***Kappa = 0.56*** |
